# Association of Lifestyle Behaviors and Cancer Risk in MetALD

**DOI:** 10.3390/cancers18162563

**Published:** 2026-08-10

**Authors:** Ryuk Jun Kwon, Yohwan Lim

**Affiliations:** 1 Family Medicine Clinic and Research Institute for Convergence of Biomedical Science and Technology, Pusan National University Yangsan Hospital, Yangsan 50612, Republic of Korea; 2 Department of Family Medicine, Pusan National University School of Medicine, Yangsan 50612, Republic of Korea; 3 Gochang-gun Public Health Center, Ministry of Health and Welfare, Gochang 77328, Republic of Korea; 4 Department of Biomedical Informatics, CHA University School of Medicine, Seongnam 13488, Republic of Korea

**Keywords:** MetALD, lifestyle behavior, diet, smoking, UK Biobank, epidemiology

## Abstract

**Background**: Metabolic and alcohol-associated liver disease (MetALD) has been associated with an increased risk of cancer. However, it remains unclear whether combinations of favorable, intermediate, and adverse lifestyle behaviors differentiate cancer risk within MetALD. **Methods**: We analyzed 25,712 participants with MetALD using UK Biobank. Smoking, moderate-to-vigorous physical activity (MVPA), and diet were classified into favorable, intermediate, and adverse categories. Favorable and adverse behavior counts ranged from 0 to 3, and a unified lifestyle profile was constructed. Cox proportional hazards models were used to analyze adjusted hazard ratios (aHRs) and 95% confidence interval (CI) for incidence of all cancers. **Results**: During 296,965 person-years of follow-up, 4416 incident cancers occurred. Compared with no favorable behaviors, those having two favorable behaviors showed significance 0.92 (95% CI, 0.84–1.00). Compared with no adverse behaviors, the aHRs were 1.06 (95% CI, 1.00–1.13), 1.15 (95% CI, 1.04–1.27), and 1.11 (95% CI, 0.84–1.47) for one, two, and three adverse behaviors, respectively (*p* for trend = 0.003). The mixed/intermediate profile was associated with higher cancer risk than the favorable profile (aHR, 1.09; 95% CI, 1.02–1.16), whereas the adverse-profile estimate was not statistically significant. Among individual lifestyle behaviors, current smoking was associated with a higher cancer risk than never smoking (aHR, 1.20; 95% CI, 1.09–1.32; *p* < 0.001). **Conclusions**: Among participants with MetALD, favorable and adverse behavior counts showed opposite adjusted trends in cancer risk. Current smoking showed the strongest association with cancer risk among the individual behaviors.

## Simple Summary

People with metabolic and alcohol-associated liver disease may have a higher risk of developing cancer, but it remains unclear how combinations of smoking, physical activity, and diet affect this risk. We studied 25,712 participants from the UKi Biobank to examine whether healthier or less healthy lifestyle patterns were related to new cancer diagnoses. During follow-up, 4416 participants developed cancer. Having more favorable behaviors tended to be associated with a lower cancer risk, whereas having more adverse behaviors tended to be associated with a higher risk, although the pattern was not consistent across every category. Current smoking showed the clearest association with increased cancer risk. Therefore, lifestyle assessment including smoking status may help identify people with metabolic and alcohol-associated liver disease who are at greater cancer risk.

## 1. Introduction

Steatotic liver disease (SLD) is one of the most common chronic liver diseases. Recently, new terminology using hepatic steatosis, cardiometabolic risk factors, and alcohol consumption was introduced as metabolic and alcohol-associated liver disease (MetALD) [1,2]. Metabolic dysfunction-associated SLD (MASLD) refers to hepatic steatosis with cardiometabolic risk factor, whereas MetALD refers to individuals with MASLD but also drink above the MASLD alcohol range [3,4]. Previous work has also emphasized that steatotic liver disease encompasses heterogeneous subtypes with different metabolic and alcohol-related characteristics [5]. Recent studies reported that MASLD and MetALD are associated with higher risks of liver cancer [6,7,8].

At the same time, modifiable lifestyles remain important factors to cancer risk. Adherence to favorable lifestyle behaviors, including avoiding smoking, maintaining physical activity, and following a healthy dietary pattern, is recommended for cancer prevention and has been associated with lower cancer incidence [9,10]. In addition, healthier behaviors reduced overall cancer incidence even with patients with metabolic abnormalities [11,12]. However, prior studies do not incorporate favorable and adverse behaviors at the same time. We therefore classified smoking, moderate-to-vigorous physical activity (MVPA), and diet into mutually exclusive favorable, intermediate, and adverse categories and evaluated their separate counts, a unified lifestyle profile, and a joint behavior matrix using favorable-by-adverse behaviors in participants with MetALD for analysis with cancer risk.

## 2. Methods

### 2.1. Eligible Participants

**Figure 1:**
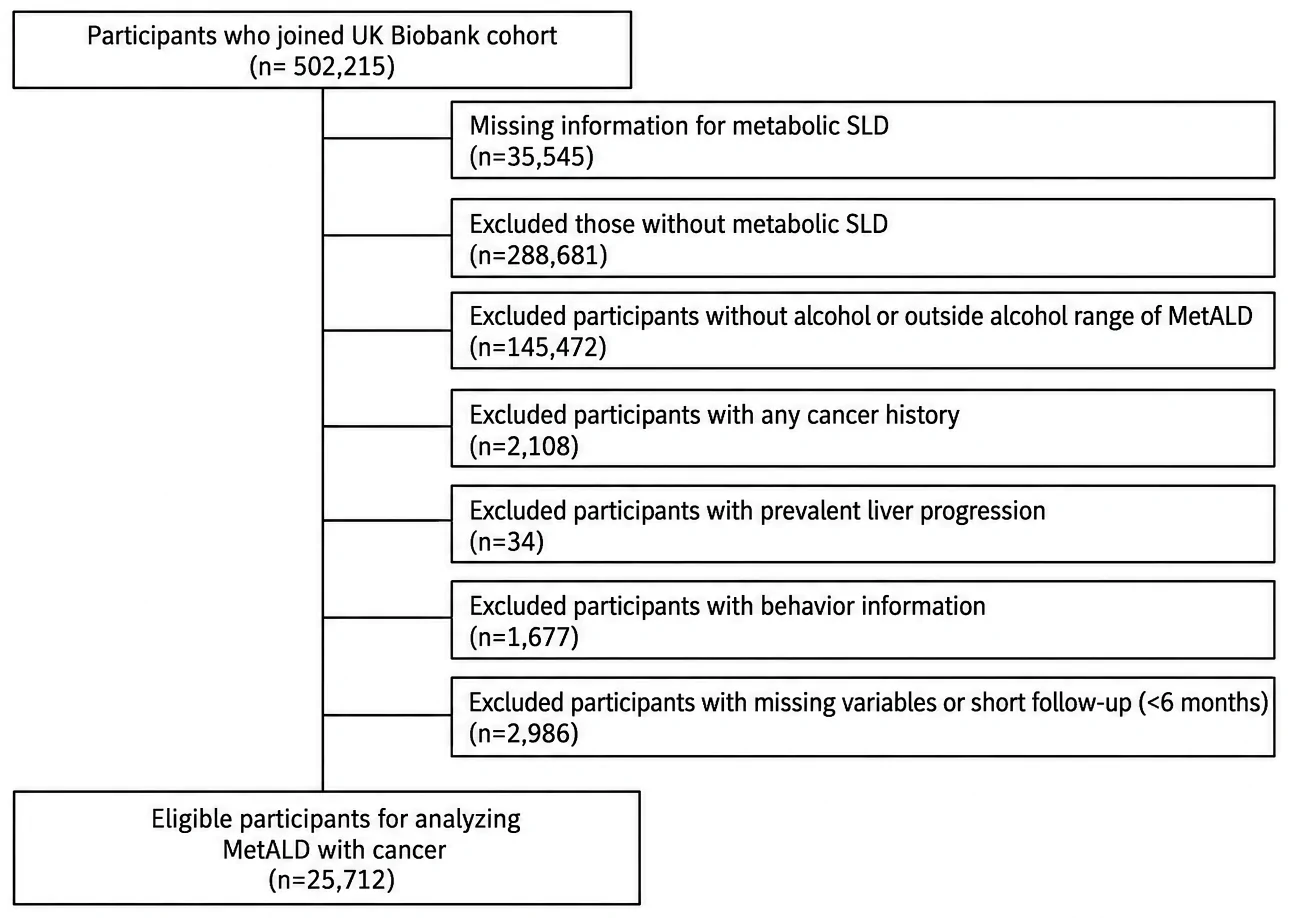
Flowdiagram for the inclusion of the study population.

We used prospective cohort data from the UK Biobank that include 500,000 adults across the United Kingdom between 2006 and 2010. They have diverse information from anthropometric measurements, laboratory data, medication histories to cancer registries, which are widely used for various medical research [13,14,15,16,17,18]. Further information of the cohort and its database is listed in previous studies [19,20]. Among the 502,215 participants, we excluded those with missing information required to define metabolic SLD (*n* = 35,545), those who did not meet the definition of metabolic SLD (*n* = 288,681), those with missing alcohol classification (*n* = 20,529), and those whose alcohol intake was outside the MetALD range (*n* = 124,943). We additionally excluded participants with prevalent cancer (*n* = 2108), prevalent liver progression (n = 34), missing baseline lifestyle information (*n* = 1677), or missing model covariates (*n* = 2841). This left 25,857 participants before the lag restriction. After excluding 145 participants who did not remain alive, cancer-free, and under observation throughout the 6-month lag period, 25,712 participants were included in the final analytic cohort (Figure 1). The UK Biobank study was approved by the North West Multi-Center Research Ethics Committee as a Research Tissue Bank (approval number: 16/NW/0274). All participants provided written informed consent before participation. This study was conducted using de-identified UK Biobank data under Application Number 106954. All procedures were performed in accordance with relevant guidelines and regulations and with the principles of the Declaration of Helsinki.

### 2.2. MetALD

MetALD was defined using metabolic SLD and alcohol intake within the MetALD range. Participants with metabolic SLD were classified as MetALD if their alcohol intake was 140 to <350 g/week in women or 210 to <420 g/week in men. Weekly alcohol intake was calculated at baseline. Alcohols were calculated by 10 g per glass of red wine, white wine, spirits, fortified wine, or other alcoholic drink, and 20 g per pint of beer or cider.

The definition for metabolic SLD was based on hepatic steatosis with at least one cardiometabolic risk factor. Hepatic steatosis was defined using the fatty liver index (FLI), calculated by triglycerides, gamma-glutamyl transferase, body mass index, and waist circumference, and steatosis was defined as FLI ≥ 60 [21]. Triglycerides were converted from mmol/L to mg/dL before FLI formula.

$$\begin{array}{c} \mathrm{FLI}=100\times e^{\left(0.953\times ln(triglycerides\;[mg/dL])+0.139\times BMI+0.718\times ln(GGT)+0.053\times WC-15.745\right)}/(1+\\ e^{\left(0.953\times ln(triglycerides\;[mg/dL])+0.139\times BMI+0.718\times ln(GGT)+0.053\times WC-15.745\right)}) \end{array}$$

Cardiometabolic risk factors were considered present if at least one risk factor was present. It included high BMI (≥23 kg/m^2^ for Asian participants and ≥25 kg/m^2^ for non-Asian participants), impaired glucose metabolism (glucose ≥ 5.6 mmol/L or use of glucose-lowering medication), higher blood pressure (systolic blood pressure ≥ 130 mmHg, diastolic blood pressure ≥ 85 mmHg, or antihypertensive treatment), or higher lipid profile (triglycerides ≥ 1.7 mmol/L, reduced HDL cholesterol, or lipid-lowering therapy) [1]. Reduced HDL cholesterol was defined as <1.0 mmol/L in men and <1.3 mmol/L in women. Participants were not classified as metabolic SLD if they had other liver disorders including viral hepatitis, alcohol-related liver disease, toxic liver disease, cholestatic liver disorders, autoimmune hepatitis, Wilson disease, and hemochromatosis. Pre-existingliver progression was defined using hospital diagnoses recorded before baseline, including chronic or advanced liver disease, portal hypertension, varices, hepatorenal syndrome, and ascites (Table S1).

### 2.3. Outcome

Cancer-registry diagnosis dates and ICD-10 codes were obtained from UK Biobank fields 40005 and 40006, respectively. The primary outcome was incidence of all cancers identified from the cancer registry using ICD-10 codes C00–C97. Codes C77–C79, which represent secondary malignant neoplasms, were excluded. For participants with multiple cancer diagnoses, the earliest diagnosis date was used. Cancers of unknown primary site coded as C80 were included in the primary outcome and excluded in a sensitivity analysis.

### 2.4. Lifestyle Behavior

We used smoking, physical activity, and diet for our lifestyle, which were classified into mutually exclusive favorable, intermediate, and adverse categories. Never smoking was classified as favorable, former smoking as intermediate, and current smoking as adverse. Weekly MVPA frequency was calculated as the sum of moderate and vigorous physical activity days. MVPA ≥ 4 days/week was classified as favorable, 2–3 days/week as intermediate, and ≤1 session/week as adverse.

Diet was evaluated using five components: vegetable, fruit, fish, processed meat, and red meat intake. A favorable diet was defined as meeting at least four favorable dietary components, whereas an adverse diet was defined as meeting at least two adverse dietary components. Participants who met neither definition were classified as having an intermediate diet. Complete information for all five dietary components was required. Further definitions for lifestyle definitions are provided in Table S2.

The favorable behavior count was calculated as the number of favorable smoking, MVPA, and diet categories and ranged from 0 to 3. The adverse behavior count was calculated similarly. We also constructed a unified lifestyle profile. Participants with at least two favorable behaviors and no adverse behaviors were classified as having a favorable profile. Those with at least two adverse behaviors and no favorable behaviors were classified as having an adverse profile. All remaining combinations were classified as mixed/intermediate.

### 2.5. Covariates

Baseline variables included age, sex, household income level, Townsend deprivation score, hypertension, diabetes, dyslipidemia, and weekly alcohol intake. Household income was categorized as lower or upper by median value. Hypertension was defined as systolic blood pressure ≥ 140 mmHg, diastolic blood pressure ≥ 90 mmHg, or use of antihypertensive medication. Diabetes was defined as fasting glucose ≥ 7.0 mmol/L or use of glucose-lowering medication. Dyslipidemia was defined as triglycerides ≥ 1.7 mmol/L, HDL cholesterol < 1.0 mmol/L in men or <1.3 mmol/L in women, or use of lipid-lowering medication. Other operational definitions of key variables are widely used in previous epidemiology studies [22,23,24,25,26,27,28,29,30].

### 2.6. Statistical Analysis

Baseline characteristics were summarized according to the favorable behavior count. Continuous variables are presented as means with standard deviations, and categorical variables as numbers with percentages. Median follow-up and its 95% confidence interval (CI) were analyzed using the reverse Kaplan–Meier method. Cancer incidence rates and 95% CI were calculated per 1000 person-years using the Poisson distribution.

Cox proportional hazards models were used to analyze adjusted hazard ratios (aHRs) and 95% CI for incidence of all cancers. For the favorable and adverse behavior-count analyses, participants with zero behaviors were used as the reference group. Model 1 was adjusted for age and sex. Model 2 was additionally adjusted for household income, hypertension, diabetes, dyslipidemia, weekly alcohol intake, and Townsend deprivation score. Linear trends were evaluated by entering each behavior count as an ordinal variable. Follow-up began 6 months after the baseline assessment and continued until the earliest occurrence of incident cancer, death, loss to follow-up, or 31 May 2022. Because assessment-center information was unavailable in the analytic dataset, 31 May 2022, the earliest common outcome-availability date, across England, Scotland, and Wales, was applied to all participants as the administrative censoring date.

The joint distribution of favorable and adverse behavior counts was evaluated using a single mutually exclusive behavior matrix. Matrix cells were labeled Hx_Uy, where x represents the number of favorable behaviors and y represents the number of adverse behaviors. For example, H0_U1 indicates 0 favorable behaviors and 1 adverse behavior, whereas H1_U2 indicates 1 favorable behavior and 2 adverse behaviors. Participants with 3 favorable behaviors and no adverse behaviors (H3_U0) were used as the reference group. The overall contribution of the matrix was evaluated using a likelihood-ratio test. Smoking, MVPA, and diet categories were also examined simultaneously in a mutually adjusted model. Potential effect modification by alcohol consumption was evaluated by adding interaction terms between alcohol intake per 50 g/week and smoking, MVPA, or diet. Interaction between the lower and upper half of the MetALD alcohol range and the unified lifestyle profile was also assessed using likelihood-ratio tests.

Sensitivity analyses applied 2-year and 5-year lag periods, excluded former smokers, excluded C80 diagnoses, and combined cancer-registry and hospital diagnoses. Death was treated as a competing event using Fine–Gray regression. Cumulative incidence functions were analyzed using the Aalen–Johansen method and compared using Gray’s test. Proportional hazards assumptions were evaluated using Schoenfeld residuals. Because age and sex contributed to violation of the global proportional hazards assumption, an additional model stratified the baseline hazard by sex and 5-year age categories. All statistical tests were two-sided, and *p* < 0.05 was considered statistically significant. Analyses were conducted using SAS version 9.4 and R version 4.5.2 (R Foundation for Statistical Computing, Vienna, Austria).

## 3. Results

### 3.1. Participant Characteristics

A total of 25,712 participants with MetALD were included in the primary analysis (Figure 1). The mean age was 56.6 years, and 19,977 participants (77.7%) were men. During 296,965 person-years of follow-up, 4416 incident cancer events occurred. The median follow-up was 12.97 years (95% CI, 12.95–12.99 years) (Table S3).

The numbers of participants with zero, one, two, and three favorable behaviors were 4135 (16.1%), 10,303 (40.1%), 8985 (34.9%), and 2289 (8.9%), respectively. The corresponding numbers with zero, one, two, and three adverse behaviors were 13,157 (51.2%), 9440 (36.7%), 2778 (10.8%), and 337 (1.3%), respectively. Other baseline characteristics are presented in Table 1.

### 3.2. Favorable Behavior Count and Incidence of All Cancers

Cancer incidence rates decreased from 15.86 per 1000 person-years among participants with no favorable behaviors to 13.20 per 1000 person-years among those with three favorable behaviors. Compared with participants with no favorable behaviors, the aHRs were 0.98 (95% CI, 0.90–1.06), 0.92 (95% CI, 0.84–1.00), and 0.89 (95% CI, 0.78–1.01) for participants with one, two, and three favorable behaviors, respectively (*p* for trend = 0.011) (Table 2).

The corresponding 10-year cumulative incidences were 15.33%, 14.94%, 13.57%, and 12.67%, respectively, with evidence of differences between the cumulative incidence functions (Gray’s test *p* = 0.006) (Table S9 and Figure S1).

### 3.3. Adverse Behavior Count and Incidence of All Cancers

Compared with participants with no adverse behaviors, the aHRs were 1.06 (95% CI, 1.00–1.13), 1.15 (95% CI, 1.04–1.27), and 1.11 (95% CI, 0.84–1.47) for those with one, two, and three adverse behaviors, respectively (*p* for trend = 0.003) (Table 3). However, cumulative incidence functions did not differ significantly across adverse behavior counts (Gray’s test *p* = 0.474) (Table S9 and Figure S2).

### 3.4. Unified Lifestyle Profile and Joint Behavior Matrix

Among the study population, 8388 participants (32.6%) had a favorable profile, 15,726 (61.2%) had a mixed/intermediate profile, and 1598 (6.2%) had an adverse profile. Compared with the favorable profile, the mixed/intermediate profile was associated with a higher risk of incident cancer (aHR, 1.09; 95% CI, 1.02–1.16; *p* = 0.010). The adverse profile had aHR of 1.12 (95% CI, 0.98–1.27; *p* = 0.100) (Table 4).

The 10-year cumulative incidences were 13.79%, 14.68%, and 13.58% in the favorable, mixed/intermediate, and adverse profiles, respectively, without a significant overall difference (Gray’s test *p* = 0.291) (Table S9 and Figure S3).

**Figure 2:**
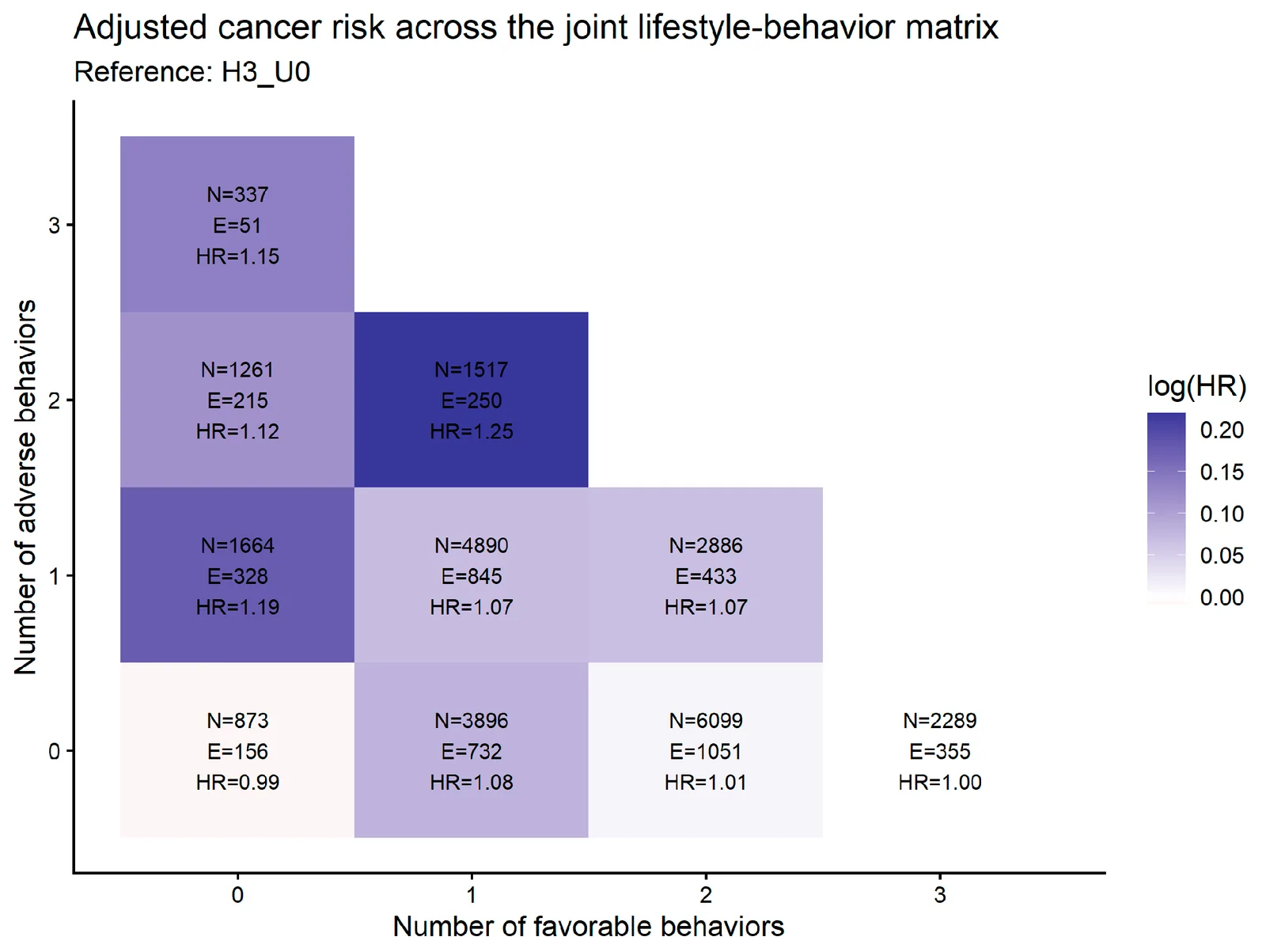
Adjusted risk of incidence of all cancers across the joint favorable and adverse lifestyle-behavior matrix. The heatmap presents adjusted hazard ratios for incidence of all cancers according to the joint number of favorable and adverse lifestyle behaviors among participants with metabolic and alcohol-associated liver disease. Favorable and adverse behaviors were defined using smoking, moderate-to-vigorous physical activity, and diet. Each cell shows the number of participants (N), number of incident cancer events (E), and adjusted hazard ratio (HR). Participants with three favorable behaviors and no adverse behaviors served as the reference group (H3_U0). Hazard ratios were estimated using Cox proportional hazard models adjusted for age, sex, household income, hypertension, diabetes, dyslipidemia, weekly alcohol intake, and Townsend deprivation score. Blank cells represent combinations that were not possible under the mutually exclusive three-level classification of each behavior. H, number of favorable behaviors; U, number of adverse behaviors; HR, hazard ratio; MetALD, metabolic and alcohol-associated liver disease.

In the joint favorable-by-adverse behavior matrix, participants with three favorable behaviors and no adverse behaviors served as the reference group. The H0_U1 group had an adjusted HR of 1.19 (95% CI, 1.03–1.39; *p* = 0.021), and the H1_U2 group had an adjusted HR of 1.25 (95% CI, 1.06–1.47; *p* = 0.008). However, the overall matrix association did not reach statistical significance (likelihood-ratio *p* = 0.068) (Figure 2 and Table S8).

### 3.5. Individual Lifestyle Behaviors and Alcohol Interactions

Current smoking was associated with a higher risk of incident cancer than never smoking (aHR, 1.20; 95% CI, 1.09–1.32; *p* < 0.001). Former smoking was not significantly associated with cancer risk (aHR, 1.03; 95% CI, 0.97–1.11). Neither the intermediate nor adverse MVPA and diet categories were independently associated with cancer risk after mutual adjustment (Table S4).

There was no statistically significant interaction between alcohol intake and smoking, MVPA, diet, or the unified lifestyle profile (Table S7).

### 3.6. Sensitivity and Competing-Risk Analyses

The association for the mixed/intermediate profile remained similar after application of a 2-year lag period (aHR, 1.09; 95% CI, 1.02–1.16), exclusion of C80 diagnoses (aHR, 1.09; 95% CI, 1.02–1.16), and inclusion of hospital cancer diagnoses (aHR, 1.07; 95% CI, 1.01–1.13). After former smokers were excluded, the aHRs were 1.14 (95% CI, 1.04–1.25) for the mixed/intermediate profile and 1.23 (95% CI, 1.02–1.48) for the adverse profile. Results from the remaining lag and outcome sensitivity analyses are presented in Table S5 and Figure S4.

In the Fine–Gray model treating death as a competing event, the subdistribution of HR was 1.08 (95% CI, 1.01–1.15; *p* = 0.021) for the mixed/intermediate profile and 1.10 (95% CI, 0.96–1.25; *p* = 0.162) for the adverse profile compared with the favorable profile (Table S6).

The proportional hazards assumption was not violated for the unified lifestyle profile itself (*p* = 0.288), although the global test was significant because of age and sex (*p* = 0.002). In the sensitivity model stratified by sex and 5-year age categories, the global proportional hazards test was no longer significant (*p* = 0.407), and the estimates remained similar (Table S10).

## 4. Discussion

In this prospective cohort of participants with MetALD, a greater number of favorable behaviors was associated with a lower risk of incidence of all cancers, whereas the adverse behavior count showed a positive adjusted trend. However, neither association followed a strictly monotonic pattern. The mixed/intermediate lifestyle profile was associated with higher cancer risk than the favorable profile, whereas the adverse profile was not statistically significant in the primary analysis. Current smoking showed the strongest association with cancer risk among the individual behaviors. Therefore, lifestyle patterns may help distinguish cancer risk among individuals with MetALD, but they do not support ranking adverse behaviors as more important than favorable behaviors.

The inverse association with favorable behaviors is generally consistent with previous studies. A UK Biobank study with metabolic syndrome found that greater adherence to a healthy lifestyle was associated with lower incident cancer and cancer mortality. Other studies combining metabolic and lifestyle risk factors also reported lower risks of overall cancers among participants with more favorable lifestyle patterns [11,12,31]. These findings support the association between lifestyle and cancer even among individuals with elevated metabolic risk. However, the associations in our study were smaller and less consistently dose-dependent. One possible explanation is that all participants in our cohort already had both metabolic dysfunction and alcohol intake within the MetALD range, resulting in a population with a relatively high and restricted baseline-risk profile. In addition, previous studies often combined a larger number of lifestyle factors into a single favorable lifestyle score than our study [11,12,32].

Our joint behavior matrix further showed that cancer risk could not be summarized by a simple comparison between favorable and adverse behavior counts. Although participants with zero favorable behaviors and one adverse behavior and those with one favorable behavior and two adverse behaviors had higher point estimates than the reference group, the overall matrix association did not reach statistical significance. Therefore, these findings should be viewed as exploratory rather than as evidence that particular combinations have definitively different risks. The absence of a strict dose–response pattern may reflect differences in the relative effects of smoking, physical activity, and diet, because each behavior contributed equally to the count despite potentially different associations with cancer. In addition, confidence intervals overlapped across several behavior-count categories, and only 337 participants had all three adverse behaviors. Therefore, the estimates were imprecise and should not be interpreted as evidence of a consistently increasing risk across all categories.

Current smoking was the clearest individual behavioral factor associated with cancer risk. This finding is consistent with carcinogenic effects of continued smoking and supports classifying former smoking separately from never smoking. Cancer risk does not immediately return to the level of never smokers after cessation and varies according to smoking intensity and time since quitting. In a large cohort, cancer risk began to decrease meaningfully after sustained smoking cessation, with larger reductions observed after longer cessation periods. Therefore, former smokers represent a heterogeneous group and should not automatically be considered to have the same risk as never smokers. Our primary classification treated former smoking as intermediate, and the sensitivity analysis excluding former smokers showed broadly consistent associations [33].

The background of MetALD is important when interpreting these findings. Previous studies have reported higher risks of gastrointestinal and liver cancers, hepatocellular carcinoma, and cirrhosis among individuals with chronic liver disease and MetALD [6,7,34]. Metabolic dysfunction and elevated alcohol intake may jointly promote hepatic steatosis, insulin resistance, chronic inflammation, and fibrosis, thereby creating a biological environment that may increase susceptibility to carcinogenesis [2,35]. Excess body fat and metabolic abnormalities are also associated with increased risks of multiple cancers [36]. Metabolic dysfunction may also accelerate atherosclerosis and cardiovascular disease, while shared pathways such as chronic inflammation and oxidative stress may contribute to carcinogenesis [37,38]. Smoking may further contribute through direct exposure to carcinogens and oxidative injury, whereas physical activity and favorable dietary patterns may improve metabolic regulation and systemic inflammation.

We did not identify statistically significant interactions between alcohol intake and smoking, physical activity, diet, or the unified lifestyle profile. Alcohol is an established carcinogen, and previous studies have reported joint effects of alcohol and tobacco for cancers [39,40]. However, our analysis was restricted to participants whose alcohol intake fell within the sex-specific MetALD range, which limited variability in alcohol exposure. In addition, cancer site specific interactions may have been diluted by the use of an all-cancer outcome, and statistical power may have been limited in smaller behavior groups. Therefore, the absence of significant interaction should be interpreted cautiously.

Several limitations should be considered. First, hepatic steatosis was identified using the fatty liver index rather than imaging, transient elastography, or histology, and some participants may therefore have been misclassified. Second, smoking, physical activity, diet, and alcohol intake were assessed at baseline and were self-reported. Changes in lifestyle during follow-up could not be incorporated, and measurement error may have weakened the observed associations. Third, detailed smoking information, including pack-years, smoking intensity, and time since cessation, was unavailable. Residual differences between former and never smokers may therefore remain despite treating former smoking as an intermediate category. Fourth, all cancers were combined into a single outcome. Because smoking, alcohol, physical activity, and diet have different associations across cancer sites, the all-cancer analysis may conceal important site-specific patterns and interactions. Fifth, cancer and other disease outcomes were identified using ICD-10 codes, and detailed tumor information was unavailable. We excluded codes C77–C79 for secondary malignant neoplasms and conducted a sensitivity analysis excluding C80 cancers of unknown primary site. Nevertheless, reliance on diagnostic codes may have resulted in underascertainment or misclassification. ICD-10-based definitions have, however, been widely used in epidemiologic studies [41,42,43,44,45,46,47]. Sixth, the complete-case analysis may have introduced selection bias because participants with missing information were excluded. The characteristics of excluded participants may differ from those of the final analytic cohort. In addition, UK Biobank participants may not fully represent the general population [20]. Seventh, residual and unmeasured confounding remains possible because of the observational design. Specific lipid-lowering therapies were not evaluated, although they may also influence cancer risk [48]. Eighth, some matrix cells and the group with three adverse behaviors contained relatively few participants.

Among participants with MetALD, favorable and adverse behavior counts showed opposite adjusted trends in incident-cancer risk, although neither association was strictly dose-dependent. Current smoking showed the most consistent association with cancer risk among the individual behaviors. The unified profile and joint matrix suggested modest differences in risk, but our findings do not show that adverse behaviors are more important than favorable behaviors. Lifestyle assessment may help identify differences in cancer risk among individuals with MetALD, although confirmation is needed in cohorts with repeated lifestyle measurements and site-specific cancer outcomes.

## Figures and Tables

**Table 1:** Baseline characteristics of participants with MetALD according to favorable behavior count.

Characteristic	Total (n = 25,712)	0 Favorable Behaviors (n = 4135)	1 Favorable Behavior (n = 10,303)	2 Favorable Behaviors (n = 8985)	3 Favorable Behaviors (n = 2289)
Age, years	56.56 (7.73)	56.75 (7.70)	56.67 (7.74)	56.46 (7.74)	56.09 (7.68)
Sex, n (%)					
Men	19,977 (77.7%)	3233 (78.2%)	8215 (79.7%)	6888 (76.7%)	1641 (71.7%)
Women	5735 (22.3%)	902 (21.8%)	2088 (20.3%)	2097 (23.3%)	648 (28.3%)
Alcohol intake, g/week	268.75 (67.71)	273.61 (67.33)	272.43 (67.49)	265.85 (67.19)	254.77 (68.95)
BMI, n (%)					
Underweight	0 (0.0%)	0 (0.0%)	0 (0.0%)	0 (0.0%)	0 (0.0%)
Normal	671 (2.6%)	132 (3.2%)	284 (2.8%)	204 (2.3%)	51 (2.2%)
Overweight	12,271 (47.7%)	1834 (44.4%)	4947 (48.0%)	4381 (48.8%)	1109 (48.4%)
Obese	12,770 (49.7%)	2169 (52.5%)	5072 (49.2%)	4400 (49.0%)	1129 (49.3%)
Hypertension, n (%)	18,729 (72.8%)	2998 (72.5%)	7478 (72.6%)	6587 (73.3%)	1666 (72.8%)
Diabetes, n (%)	1687 (6.6%)	353 (8.5%)	677 (6.6%)	537 (6.0%)	120 (5.2%)
Dyslipidemia, n (%)	20,307 (79.0%)	3394 (82.1%)	8186 (79.5%)	7000 (77.9%)	1727 (75.4%)
Diet favorable score, n (%)					
0	311 (1.2%)	87 (2.1%)	172 (1.7%)	52 (0.6%)	0 (0.0%)
1	2008 (7.8%)	609 (14.7%)	1005 (9.8%)	394 (4.4%)	0 (0.0%)
2	5299 (20.6%)	1416 (34.2%)	2741 (26.6%)	1142 (12.7%)	0 (0.0%)
3	8147 (31.7%)	2023 (48.9%)	4302 (41.8%)	1822 (20.3%)	0 (0.0%)
4	7663 (29.8%)	0 (0.0%)	1632 (15.8%)	4254 (47.3%)	1777 (77.6%)
5	2284 (8.9%)	0 (0.0%)	451 (4.4%)	1321 (14.7%)	512 (22.4%)
Income level, n (%)					
Upper half	7516 (29.2%)	1194 (28.9%)	2890 (28.1%)	2722 (30.3%)	710 (31.0%)
Lower half	18,196 (70.8%)	2941 (71.1%)	7413 (71.9%)	6263 (69.7%)	1579 (69.0%)
Cigarette smoking, n (%)					
Current smoker	3646 (14.2%)	1058 (25.6%)	1814 (17.6%)	774 (8.6%)	0 (0.0%)
Former smoker	12,772 (49.7%)	3077 (74.4%)	6115 (59.4%)	3580 (39.8%)	0 (0.0%)
Never smoker	9294 (36.1%)	0 (0.0%)	2374 (23.0%)	4631 (51.5%)	2289 (100.0%)
Townsend deprivation score	−1.34 (3.01)	−1.14 (3.12)	−1.26 (3.03)	−1.48 (2.96)	−1.54 (2.89)

Continuous variables are presented as mean (standard deviation), and categorical variables as n (%). Favorable behaviors were never smoking, MVPA ≥ 4 days/week, and a favorable diet. Former smoking and intermediate MVPA or diet categories did not contribute to the favorable count. BMI categories used ethnicity-specific cutoffs. Acronyms: MetALD, metabolic and alcohol-associated liver disease; MVPA, moderate-to-vigorous physical activity; BMI, body mass index.

**Table 2:** Incidence rates and hazard ratios for incidence of all cancers according to favorable behavior count.

Favorable Behavior	Event/Total	Person-Years	IR per 1000 PY (95% CI)	Model 1 HR (95% CI)	*p* for Trend	Model 2 HR (95% CI)	*p* for Trend
0 favorable behaviors	750/4135	47,296	15.86 (14.74–17.03)	1.00 (Ref)	0.009	1.00 (Ref)	0.011
1 favorable behavior	1827/10,303	118,126	15.47 (14.77–16.19)	0.98 (0.90–1.06)		0.98 (0.90–1.06)	
2 favorable behaviors	1484/8985	104,649	14.18 (13.47–14.92)	0.91 (0.84–1.00) *		0.92 (0.84–1.00) *	
3 favorable behaviors	355/2289	26,894	13.20 (11.86–14.65)	0.89 (0.78–1.01)		0.89 (0.78–1.01)	

Favorable behaviors were never smoking, MVPA ≥ 4 days/week, and a favorable diet (≥4 favorable diet components). Model 1 adjusted for age and sex. Model 2 additionally adjusted for household income, hypertension, diabetes, dyslipidemia, weekly alcohol intake, and Townsend deprivation score. *p* for trend was estimated by entering the behavior count as an ordinal variable. * *p* < 0.05. Acronyms: PY, person-years; IR, incidence rate; HR, hazard ratio; CI, confidence interval.

**Table 3:** Incidence rates and hazard ratios for incidence of all cancers according to adverse behavior count.

Adverse Behavior	Event/Total	Person-Years	IR per 1000 PY (95% CI)	Model 1 HR (95% CI)	*p* for Trend	Model 2 HR (95% CI)	*p* for Trend
0 adverse behaviors	2294/13,157	151,948	15.10 (14.49–15.73)	1.00 (Ref)	0.003	1.00 (Ref)	0.003
1 adverse behavior	1606/9440	108,892	14.75 (14.04–15.49)	1.06 (1.00–1.13)		1.06 (1.00–1.13)	
2 adverse behaviors	465/2778	32,254	14.42 (13.14–15.79)	1.15 (1.04–1.27) **		1.15 (1.04–1.27) **	
3 adverse behaviors	51/337	3870	13.18 (9.81–17.33)	1.11 (0.84–1.47)		1.11 (0.84–1.47)	

Adverse behaviors were current smoking, MVPA ≤ 1 session/week, and an adverse diet (≥2 adverse diet components). Model 1 adjusted for age and sex. Model 2 additionally adjusted for household income, hypertension, diabetes, dyslipidemia, weekly alcohol intake, and Townsend deprivation score. *p* for trend was estimated by entering the behavior count as an ordinal variable. * *p* < 0.05; ** *p* < 0.01. Acronyms: PY, person-years; IR, incidence rate; HR, hazard ratio; CI, confidence interval.

**Table 4:** Association between the unified lifestyle profile and incidence of all cancers.

Unified Lifestyle Profile	Event/Total	Person-Years	IR per 1000 PY (95% CI)	Adjusted HR (95% CI)	*p* Value	10-Year Cumulative Incidence
Favorable profile	1406/8388	97,581	14.41 (13.67–15.18)	1.00 (Ref)	—	13.79%
Mixed/intermediate profile	2744/15,726	180,862	15.17 (14.61–15.75)	1.09 (1.02–1.16) *	0.010	14.68%
Adverse profile	266/1598	18,522	14.36 (12.69–16.19)	1.12 (0.98–1.27)	0.100	13.58%

The favorable profile was defined as at least two favorable behaviors and no adverse behavior. The adverse profile was defined as at least two adverse behaviors and no favorable behavior; all other combinations were classified as mixed/intermediate. Adjusted hazard ratios were estimated after adjustment for age, sex, household income, hypertension, diabetes, dyslipidemia, weekly alcohol intake, and Townsend deprivation score. Ten-year cumulative incidence was estimated using the Aalen–Johansen method with death treated as a competing event. * *p* < 0.05. Acronyms: PY, person-years; IR, incidence rate; HR, hazard ratio; CI, confidence interval.

## Data Availability

The data is only available to authorized researchers in UK Biobank.

## Supplementary Materials

The following supporting information can be downloaded at https://www.mdpi.com/article/10.3390/cancers18162563/s1, Table S1: List of liver diseases by ICD-10 codes; Table S2: Operational definitions of favorable, intermediate, and adverse lifestyle behaviors; Table S3: Analytic cohorts used in the primary and sensitivity analyses; Table S4: Associations of individual lifestyle behaviors with incident all cancer; Table S5: Unified lifestyle profile and sensitivity analyses; Table S6: Competing-risk analysis for the unified lifestyle profile; Table S7: Interactions between alcohol intake and lifestyle behaviors; Table S8: Adjusted hazard ratios across the joint favorable-by-adverse behavior matrix; Table S9. 10-year cumulative incidence of all cancer; Table S10: Proportional-hazards assumption tests for the unified lifestyle profile models; Figure S1: Cumulative incidence of all cancer according to favorable behavior count; Figure S2: Cumulative incidence of all cancer according to adverse behavior count; Figure S3: Cumulative incidence of all cancer according to the unified lifestyle profile; Figure S4: Sensitivity analyses for the unified lifestyle profile.

## References

[1] Rinella M.E., Lazarus J.V., Ratziu V., Francque S.M., Sanyal A.J., Kanwal F., Romero D., Abdelmalek M.F., Anstee Q.M., Arab J.P. (2023). A multisociety Delphi consensus statement on new fatty liver disease nomenclature. Hepatology.

[2] Leal-Lassalle H., Estévez-Vázquez O., Cubero F.J., Nevzorova Y.A. (2025). Metabolic and alcohol-associated liver disease (MetALD): A representation of duality. npj Gut Liver.

[3] Liu Z., Lin C., Suo C., Zhao R., Jin L., Zhang T., Chen X. (2022). Metabolic dysfunction–associated fatty liver disease and the risk of 24 specific cancers. Metabolism.

[4] Tacke F., Horn P., Wong V.W.-S., Ratziu V., Bugianesi E., Francque S., Zelber-Sagi S., Valenti L., Roden M., Schick F. (2024). EASL–EASD–EASO Clinical Practice Guidelines on the management of metabolic dysfunction-associated steatotic liver disease (MASLD): Executive Summary. Diabetologia.

[5] Jeong S., Lim Y., Lee S.K., Han H.W. (2023). Letter regarding “Non-alcoholic fatty liver disease: Definition and subtypes”. Clin. Mol. Hepatol..

[6] Park Y., Jung J., Han S., Kim G.-A. (2024). Metabolic dysfunction-associated steatotic liver disease and MetALD increases the risk of liver cancer and gastrointestinal cancer: A nationwide cohort study. Aliment. Pharmacol. Ther..

[7] Kim G.A., Jeong S., Jang H., Lee D.H., Joo S.K., Kim W. (2024). Metabolic Dysfunction-Associated Steatotic Liver Disease and Metabolic Dysfunction-Associated Steatotic Liver Disease with Increased Alcohol Intake Increase the Risk of Developing Hepatocellular Carcinoma and Incident or Decompensated Cirrhosis: A Korean Nationwide Study. Liver Cancer.

[8] Kwon R.J., Lim Y. (2026). Association of metabolic dysfunction-associated steatotic liver disease trajectories with incident liver cancer: A UK Biobank cohort study. Front. Endocrinol..

[9] Rock C.L., Thomson C., Gansler T., Gapstur S.M., McCullough M.L., Patel A.V., Andrews K.S., Bandera E.V., Spees C.K., Robien K. (2020). American Cancer Society guideline for diet and physical activity for cancer prevention. CA Cancer J. Clin..

[10] Clinton S.K., Giovannucci E.L., Hursting S.D. (2020). The World Cancer Research Fund/American Institute for Cancer Research Third Expert Report on Diet, Nutrition, Physical Activity, and Cancer: Impact and Future Directions. J. Nutr..

[11] Wu E., Ni J.T., Zhu Z.H., Xu H.Q., Tao L., Xie T. (2022). Association of a Healthy Lifestyle with All-Cause, Cause-Specific Mortality and Incident Cancer among Individuals with Metabolic Syndrome: A Prospective Cohort Study in UK Biobank. Int. J. Environ. Res. Public Health.

[12] Lin F., Hu W., Yang C., Cheng B., Chen H., Li J., Zhu H., Zhang H., Yuan X., Ren X. (2025). Associations of combined lifestyle and metabolic risks with cancer incidence in the UK biobank study. BMC Cancer.

[13] Tyrrell J., Mulugeta A., Wood A.R., Zhou A., Beaumont R.N., Tuke M.A., Jones S.E., Ruth K.S., Yaghootkar H., Sharp S. (2019). Using genetics to understand the causal influence of higher BMI on depression. Int. J. Epidemiol..

[14] Min J., Cao Z., Chen H., Wang X., Xu C. (2024). Trajectories of depressive symptoms and risk of cardiovascular disease, cancer and mortality: A prospective cohort study. Gen. Psychiatry.

[15] Kwak T., Kang E.S., Song J., Gökbulut B., Jang S., Kang M., Lee J., Kong S., Kim Y., Park J.-H. (2026). Regression of Metabolic Dysfunction–Associated Steatotic Liver Disease in Older Adults: Short- and Long-Term Prediction Models in the NHIS-Senior and UK Biobank Cohorts. Nutr. Metab. Cardiovasc. Dis..

[16] Lim Y. (2026). Night-Shift Intensity, Duration, Cardiovascular Susceptibility and Incident Cardiovascular Disease: UK Biobank Study. Am. J. Med..

[17] Lim Y., Kwon R. (2026). Tu1405 Dynamic Colonoscopy Sequences and 10-Year Colorectal Cancer Risk: Preliminary Evidence From a Nationwide Cohort Using Marginal Structural Models. Gastroenterology.

[18] Sudlow C., Gallacher J., Allen N., Beral V., Burton P., Danesh J., Downey P., Elliott P., Green J., Landray M. (2015). UK biobank: An open access resource for identifying the causes of a wide range of complex diseases of middle and old age. PLoS Med..

[19] Lim Y., Lee S.K. (2026). Association of organ dysfunction trajectories and major adverse cardiovascular events using clinical obesity in UK Biobank. Front. Endocrinol..

[20] Allen N.E., Lacey B., Lawlor D.A., Pell J.P., Gallacher J., Smeeth L., Elliott P., Matthews P.M., Lyons R.A., Whetton A.D. (2024). Prospective study design and data analysis in UK Biobank. Sci. Transl. Med..

[21] Bedogni G., Bellentani S., Miglioli L., Masutti F., Passalacqua M., Castiglione A., Tiribelli C. (2006). The Fatty Liver Index: A simple and accurate predictor of hepatic steatosis in the general population. BMC Gastroenterol..

[22] Lim Y., Choi Y., Kang E., Jeong Y., Park J., Han H.W. (2024). Association between short- and medium-term exposure to air pollutants and depressive episode using comprehensive air quality index among the population in South Korea. J. Affect. Disord..

[23] Lim Y., Lee M.H., Jeong S., Han H.W. (2023). Association of Physical Activity With SARS-CoV-2 Infection and Severe Clinical Outcomes Among Patients in South Korea. JAMA Netw. Open.

[24] Lim Y. (2025). Longitudinal association between consecutive moderate-to-vigorous physical activity and the risk of depression among depressed and non-depressed participants: A nationally representative cohort study. J. Affect. Disord..

[25] Lee S.-J., Yoon S.-S., Lee M.-H., Kim H.-J., Lim Y., Park H., Park S.J., Jeong S., Han H.-W. (2022). Health-Screening-Based Chronic Obstructive Pulmonary Disease and Its Effect on Cardiovascular Disease Risk. J. Clin. Med..

[26] Jeong S., Lim Y.H., Lee M.H., Han H.W. (2024). Predicted Body Composition Against COVID-19: A Potential Digital Health Strategy. MEDINFO 2023—The Future Is Accessible.

[27] Kim H.J., Lim Y., Yoon S.S., Lee S.J., Lee M.H., Park H., Park S.J., Jeong S., Han H.W. (2022). Development and validation of a nonalcoholic fatty liver disease-based self-diagnosis tool for diabetes. Ann. Transl. Med..

[28] Lim Y., Lee M.H., Lee S.K., Jeong S., Han H.W. (2023). Increased Estimated GFR Is Negatively Associated With the Risk of SARS-CoV-2 Infection and Severe COVID-19 Within Normal to Mildly Decreased Levels: Nested Case-Control Study. J. Korean Med. Sci..

[29] Peng Y., Wang P., Liu F., Wang X., Si C., Gong J., Zhou H., Gu J., Qin A., Song W. (2025). Metabolic dysfunction-associated steatotic liver disease and cancer risk: A cohort study. Diabetes Obes. Metab..

[30] Lim Y., Kim B.C., Yoon S.S., Kim H.J., Lee S.J., Lee M.H., Kim J.H., Park S.J., Jeong S., Han H.W. (2023). Inverse association between changes in systolic and diastolic blood pressure and risk of depression: A nationally representative cohort study. J. Affect. Disord..

[31] Xie P., Wu S., Kuo Z., Tian H., He Q., Li Y., Mi N., Hu L., Zhao H., Li W. (2023). Association of modifiable lifestyle with colorectal cancer incidence and mortality according to metabolic status: Prospective cohort study. Front. Oncol..

[32] Luu N.M., Bui T.T., Tran T.P.T., Nguyen T.H.T., Oh J.K. (2023). Combinations of lifestyle behaviors and cancer risk among Korean adults. Sci. Rep..

[33] Park E., Kang H.Y., Lim M.K., Kim B., Oh J.K. (2024). Cancer Risk Following Smoking Cessation in Korea. JAMA Netw. Open.

[34] Yoon S.S., Lee S.J., Kim H.J., Lee M.H., Lim Y.H., Jeong S., Jung S.S., Han H.W. (2022). Association of Weight Change with Risk of Primary Liver Cancer in Middle-Aged Men with Chronic Liver Disease.

[35] Gao B., Arab J.P., Liangpunsakul S., Ding W.-X., Szabo G., Mehal W., Wang H., He Y., Stärkel P., Llorente C. (2025). Metabolic dysfunction and alcohol-associated liver disease (MetALD). eGastroenterology.

[36] Lauby-Secretan B., Scoccianti C., Loomis D., Grosse Y., Bianchini F., Straif K. (2016). Body Fatness and Cancer--Viewpoint of the IARC Working Group. N. Engl. J. Med..

[37] Ridker P.M., MacFadyen J.G., Thuren T., Everett B.M., Libby P., Glynn R.J. (2017). Effect of interleukin-1β inhibition with canakinumab on incident lung cancer in patients with atherosclerosis: Exploratory results from a randomised, double-blind, placebo-controlled trial. Lancet.

[38] Lim Y., Jeong S., Hong M., Han H.W. (2023). Non-Alcoholic Fatty Liver Disease, Atherosclerosis, and Cardiovascular Disease in Asia. Rev. Cardiovasc. Med..

[39] Hashibe M., Brennan P., Chuang S.C., Boccia S., Castellsague X., Chen C., Curado M.P., Dal Maso L., Daudt A.W., Fabianova E. (2009). Interaction between tobacco and alcohol use and the risk of head and neck cancer: Pooled analysis in the International Head and Neck Cancer Epidemiology Consortium. Cancer Epidemiol. Biomark. Prev..

[40] Rumgay H., Shield K., Charvat H., Ferrari P., Sornpaisarn B., Obot I., Islami F., Lemmens V.E.P.P., Rehm J., Soerjomataram I. (2021). Global burden of cancer in 2020 attributable to alcohol consumption: A population-based study. Lancet Oncol..

[41] Huang H., Liu Z., Guo Y., Zeng Y., Shen S., Xu C. (2024). Long-term Use of Proton Pump Inhibitors is Associated With An Increased Risk of Nonalcoholic Fatty Liver Disease. J. Clin. Gastroenterol..

[42] Lee S.K., Lim Y., Jeong S., Han H.W. (2024). COVID-19-related cardiovascular disease risk due to weight gain: A nationwide cohort study. Eur. J. Med. Res..

[43] Yoon S.S., Lim Y., Jeong S., Han H.W. (2023). Association of weight changes with SARS-CoV-2 infection and severe COVID-19 outcomes: A nationwide retrospective cohort study. J. Infect. Public Health.

[44] Lim Y., Kim H.J., Yoon S.S., Lee S.J., Lee M.H., Park H., Park S.J., Jeong S., Han H.W. (2023). Exercise Frequency Reduction Is Associated With Higher Risk of Infection in Newly Diagnosed Diabetes: A Nationally Representative Cohort Study. J. Korean Med. Sci..

[45] Lee S.K., Lim Y., Jeong S., Han H.W. (2023). Poorer outcomes following COVID-19 infection for patients with depression: A cohort analysis in South Korea. Ann. Acad. Med. Singap..

[46] Lim Y.H., Lee M.H., Kim G.H., Pyun D.B., Jeong S., Han H.W. (2024). Moderate-to-Vigorous Physical Activity Changes with the Risk of SARS-CoV-2 Infection: A Nested Case-Control Study. Stud. Health Technol. Inform..

[47] Park J.-H., Kong S., Lim Y., Oh Y., Chawanid P., Gökbulut B., Kim J.-W., Park S.J., Kim H.J., Cheon S. (2026). Longitudinal changes in cardiorespiratory fitness and risk of depressive and anxiety disorders in a nationwide cohort of 7 million participants. Sci. Rep..

[48] Lim Y., Kim E., Hwang E., Kim K., Son Y., Lee S.Y., Cho Y.H., Park E.J., Lee Y., Lee S.R. (2025). Real-world evidence on the additional cancer risk reduction of ezetimibe: A 10-year nationwide retrospective cohort study emulating a target trial. J. Cancer Res. Clin. Oncol..

