# Supplementary material for: Association of Lifestyle Behaviors and Cancer Risk in MetALD
Source: Cancers (Basel). 2026 Aug 10;18(16):2563. doi: 10.3390/cancers18162563 (PMC13511256; doi:10.3390/cancers18162563)
Supplement: Supplementary file 1 [file cancers-18-02563-s001.zip › cancers-4447226-supplementary.pdf]

Supplementary Material

"Association of Lifestyle Behaviors and Cancer Risk in MetALD"

Table S1. List of liver diseases by ICD-10 codes

Table S2. Operational definitions of favorable, intermediate, and adverse lifestyle behaviors

Table S3. Analytic cohorts used in the primary and sensitivity analyses

Table S4. Associations of individual lifestyle behaviors with incident all cancer

Table S5. Unified lifestyle profile and sensitivity analyses

Table S6. Competing-risk analysis for the unified lifestyle profile

Table S7. Interactions between alcohol intake and lifestyle behaviors

Table S8. Adjusted hazard ratios across the joint favorable-by-adverse behavior matrix

Table S9. 10-year cumulative incidence of all cancer

Table S10. Proportional-hazards assumption tests for the unified lifestyle profile models

Figure S1. Cumulative incidence of all cancer according to favorable behavior count.

Figure S2. Cumulative incidence of all cancer according to adverse behavior count.

Figure S3. Cumulative incidence of all cancer according to the unified lifestyle profile.

Figure S4. Sensitivity analyses for the unified lifestyle profile.

Table S1. List of liver diseases by ICD-10 codes

Other liver diseases excluded when defining metabolic SLD	ICD-10 codes
Viral hepatitis	B15-B19
Alcohol-related liver disease	K70
Toxic liver disease	K71
Cholestatic liver diseases	K74.3-K74.5
Autoimmune hepatitis	K75.4
Wilson disease	E83.0
Hemochromatosis	E83.1
**Prevalent chronic or advanced liver disease excluded at baseline**	
Alcohol-related liver disease	K70
Toxic liver disease	K71
Hepatic failure	K72
Chronic hepatitis	K73
Fibrosis and cirrhosis	K74
Other inflammatory liver diseases	K75
Portal hypertension	K76.6
Hepatorenal syndrome	K76.7
Esophageal varices	I85
Gastric varices	I86.4
Ascites	R18

Prevalent chronic or advanced liver disease was defined as any listed diagnosis recorded on or before the baseline assessment date. The earliest hospital diagnosis date was used.

Acronym: ICD-10, International Statistical Classification of Diseases and Related Health Problems, 10th Revision.

Table S2. Operational definitions of favorable, intermediate, and adverse lifestyle behaviors

Behavior and UK Biobank field(s)	Favorable	Intermediate	Adverse	Construction and missing-data rule
Smoking (p20116_i0)	Never smoker	Former smoker	Current smoker	Participants without a valid baseline smoking category were treated as missing.
MVPA (p884_i0 and p904_i0)	≥4 days/week	2-3 days/week	≤1 session/week	Weekly MVPA frequency was calculated by summing moderate and vigorous activity days. When only one component was available, the available frequency was used.
Vegetables (p1289_i0 and p1299_i0)	≥4 tablespoons/day	2 to <4 tablespoons/day	<2 tablespoons/day	Cooked and raw vegetable intake were summed.
Fruit (p1309_i0 and p1319_i0)	≥2 pieces/day	1 to <2 pieces/day	<1 piece/day	Fresh and dried fruit intake were summed.
Fish (p1329_i0 and p1339_i0)	At least once/week	—	Less than once/week	The higher reported frequency of oily or non-oily fish intake was used.
Processed meat (p1349_i0)	Never or <1 time/week	Once/week	≥2-4 times/week	Baseline processed-meat frequency was used.
Red meat (p1369_i0, p1379_i0, and p1389_i0)	Never, <1 time/week, or once/week	—	≥2-4 times/week	The highest frequency among beef, lamb/mutton, and pork intake was used.
Overall diet	≥4 favorable diet components	All other complete combinations	≥2 adverse diet components	Complete information for all five diet components was required.
Behavior counts and unified profile	Each favorable behavior contributed 1 to the favorable count	Mixed/intermediate profile: all remaining combinations	Each adverse behavior contributed 1 to the adverse count	Favorable profile: ≥2 favorable behaviors and no adverse behavior. Adverse profile: ≥2 adverse behaviors and no favorable behavior.

MVPA, moderate-to-vigorous physical activity. UK Biobank special response codes and invalid values were treated as missing before applying the thresholds.

Table S3. Analytic cohorts used in the primary and sensitivity analyses

Analysis	Definition	N	Cancer events	Competing deaths	Person-years
Primary analysis	6-month lag; cancer-registry outcome	25,712	4,416	1,185	296,964.9
Two-year lag	24-month lag; cancer-registry outcome	25,223	3,976	1,136	258,776.3
Five-year lag	60-month lag; cancer-registry outcome	24,021	2,912	998	184,864.8
Cancer of unknown primary excluded	6-month lag; ICD-10 C80 excluded	25,712	4,404	1,196	296,980.3
Registry plus hospital diagnoses	6-month lag; cancer-registry and hospital diagnoses	25,702	5,204	1,053	294,801.2

Median follow-up in the primary cohort was 12.97 years (95% CI 12.95-12.99) using the reverse Kaplan-Meier method. The primary analysis used a 6-month lag after baseline. Death before incident cancer was treated as a competing event in competing-risk analyses.

Table S4. Associations of individual lifestyle behaviors with incident all cancer

Behavior	Category	Adjusted HR (95% CI)	P value
Smoking	Never smoker	1.00 (Ref)	—
	Former smoker	1.03 (0.97-1.11)	0.329
	Current smoker	1.20 (1.09-1.32)	<0.001
MVPA	Favorable	1.00 (Ref)	—
	Intermediate	1.02 (0.95-1.11)	0.570
	Adverse	1.02 (0.95-1.10)	0.589
Diet	Favorable	1.00 (Ref)	—
	Intermediate	1.04 (0.97-1.12)	0.258
	Adverse	1.06 (0.98-1.14)	0.127

The model simultaneously included smoking, MVPA, and diet and was additionally adjusted for age, sex, household income, hypertension, diabetes, dyslipidemia, weekly alcohol intake, and Townsend deprivation score. HR, hazard ratio; CI, confidence interval.

Table S5. Unified lifestyle profile and sensitivity analyses

Analysis	N	Events	Favorable profile	Mixed/intermediate profile	Adverse profile
Primary analysis	25,712	4,416	1.00 (Ref)	1.09 (1.02-1.16); P=0.010	1.12 (0.98-1.27); P=0.100
Two-year lag	25,223	3,976	1.00 (Ref)	1.09 (1.02-1.16); P=0.015	1.12 (0.97-1.29); P=0.110
Five-year lag	24,021	2,912	1.00 (Ref)	1.08 (1.00-1.17); P=0.052	1.15 (0.98-1.35); P=0.085
C80 excluded	25,712	4,404	1.00 (Ref)	1.09 (1.02-1.16); P=0.013	1.12 (0.98-1.28); P=0.094
Registry plus hospital diagnoses	25,702	5,204	1.00 (Ref)	1.07 (1.01-1.13); P=0.028	1.15 (1.02-1.30); P=0.022
Former smokers excluded	12,940	1,999	1.00 (Ref)	1.14 (1.04-1.25); P=0.007	1.23 (1.02-1.48); P=0.029
Sex- and age-stratified model	25,712	4,416	1.00 (Ref)	1.08 (1.01-1.15); P=0.016	1.10 (0.96-1.25); P=0.175

The favorable profile was defined as at least two favorable behaviors and no adverse behavior. The adverse profile was defined as at least two adverse behaviors and no favorable behavior; all remaining combinations were classified as mixed/intermediate. Models were adjusted for age, sex, household income, hypertension, diabetes, dyslipidemia, weekly alcohol intake, and Townsend deprivation score. The stratified model allowed the baseline hazard to vary by sex and 5-year age category.

Table S6. Competing-risk analysis for the unified lifestyle profile

Unified lifestyle profile	Subdistribution HR (95% CI)	P value
Favorable profile	1.00 (Ref)	—
Mixed/intermediate profile	1.08 (1.01-1.15)	0.021
Adverse profile	1.10 (0.96-1.25)	0.162

Fine-Gray regression treated death before incident cancer as a competing event (N=25,712; cancer events=4,416; competing deaths=1,185). The model was adjusted for age, sex, household income, hypertension, diabetes, dyslipidemia, weekly alcohol intake, and Townsend deprivation score.

Table S7. Interactions between alcohol intake and lifestyle behaviors

Interaction	P for interaction
Alcohol per 50 g/week × smoking	0.144
Alcohol per 50 g/week × MVPA	0.059
Alcohol per 50 g/week × diet	0.874
Alcohol-range half × unified profile	0.247

Interaction P values were obtained using likelihood-ratio tests comparing models with and without the relevant interaction term. Alcohol intake was modeled per 50 g/week for the smoking, MVPA, and diet interactions.

Table S8. Adjusted hazard ratios across the joint favorable-by-adverse behavior matrix

Favorable behaviors	Adverse behaviors	N	Events	Adjusted HR (95% CI)	P value
0	0	873	156	0.99 (0.82-1.20)	0.930
0	1	1,664	328	1.19 (1.03-1.39)	0.021
0	2	1,261	215	1.12 (0.95-1.33)	0.178
0	3	337	51	1.15 (0.85-1.54)	0.363
1	0	3,896	732	1.08 (0.96-1.23)	0.209
1	1	4,890	845	1.07 (0.95-1.21)	0.270
1	2	1,517	250	1.25 (1.06-1.47)	0.008
2	0	6,099	1,051	1.01 (0.90-1.14)	0.856
2	1	2,886	433	1.07 (0.93-1.23)	0.347
3	0	2,289	355	1.00 (Ref)	—

The reference group was H3_U0 (three favorable behaviors and no adverse behavior). Overall likelihood-ratio test P=0.068. Models were adjusted for age, sex, household income, hypertension, diabetes, dyslipidemia, weekly alcohol intake, and Townsend deprivation score. H, favorable behavior count; U, adverse behavior count.

Table S9. 10-year cumulative incidence of all cancer

Exposure	Group	10-year cumulative incidence	Gray-test P value
Favorable behavior count	0 favorable behaviors	15.33%	0.006
	1 favorable behavior	14.94%	
	2 favorable behaviors	13.57%	
	3 favorable behaviors	12.67%	
Adverse behavior count	0 adverse behaviors	14.65%	0.474
	1 adverse behavior	14.15%	
	2 adverse behaviors	13.53%	
	3 adverse behaviors	12.46%	
Unified lifestyle profile	Favorable profile	13.79%	0.291
	Mixed/intermediate profile	14.68%	
	Adverse profile	13.58%	

Cumulative incidence was estimated using the Aalen-Johansen method with death before incident cancer treated as a competing event.

Table S10. Proportional-hazards assumption tests for the unified lifestyle profile models

Model	Test component	Chi-square (df)	P value
Primary Model 2	Unified lifestyle profile	2.493 (2)	0.288
Primary Model 2	Age	9.757 (1)	0.002
Primary Model 2	Sex	9.539 (1)	0.002
Primary Model 2	Global test	28.447 (10)	0.002
Sex- and age-stratified Model 2	Unified lifestyle profile	2.847 (2)	0.241
Sex- and age-stratified Model 2	Global test	8.273 (8)	0.407

The primary exposure did not violate the proportional-hazards assumption. Because age, sex, and the global test indicated non-proportionality in the primary model, a sensitivity model stratified by sex and 5-year age category was fitted; the exposure-specific and global tests were then non-significant.

Figure S1. Cumulative incidence of all cancer according to favorable behavior count.

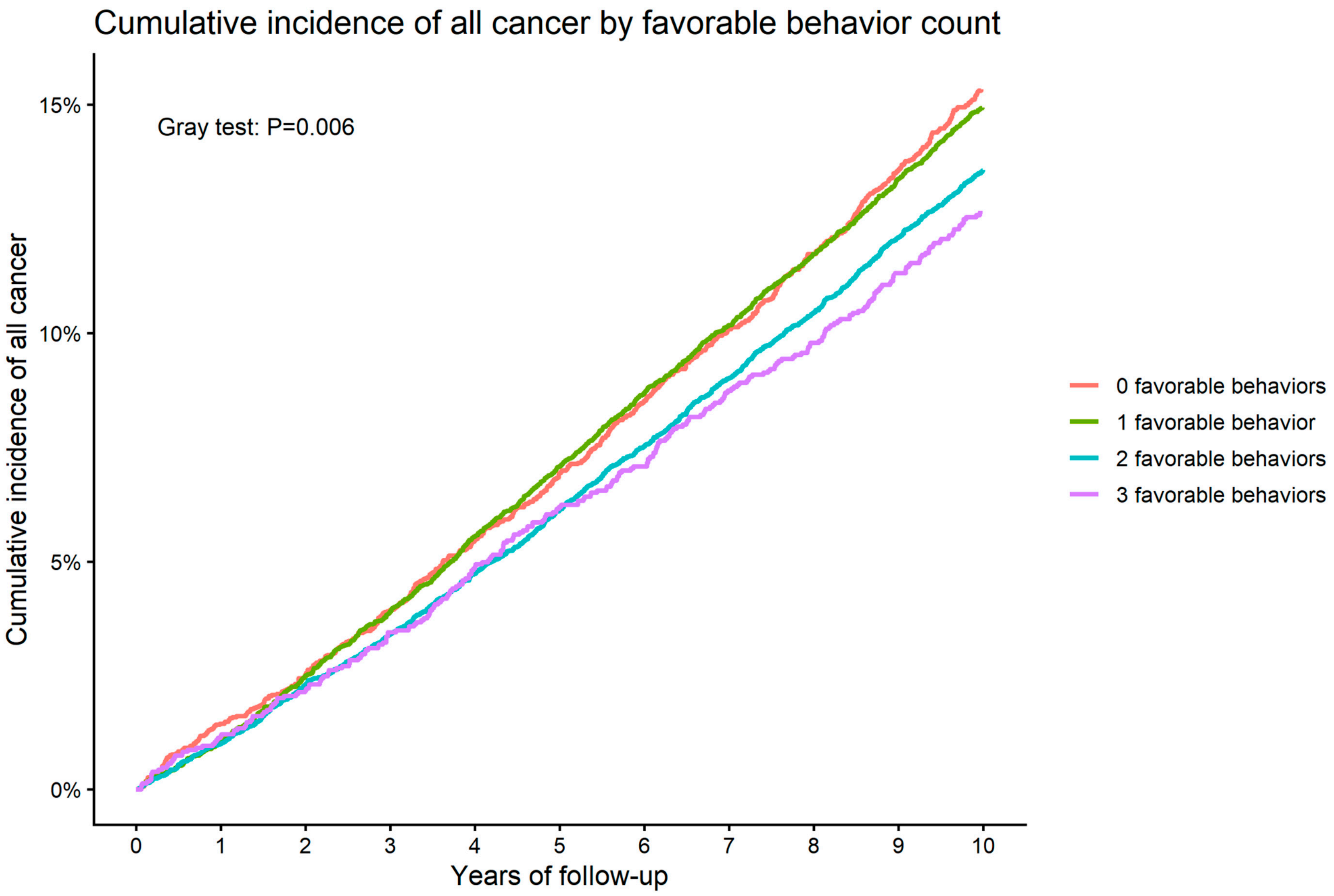

Curves were estimated using the Aalen-Johansen method with death treated as a competing event. The Gray-test P value compares cumulative-incidence functions across the four groups.

Figure S2. Cumulative incidence of all cancer according to adverse behavior count.

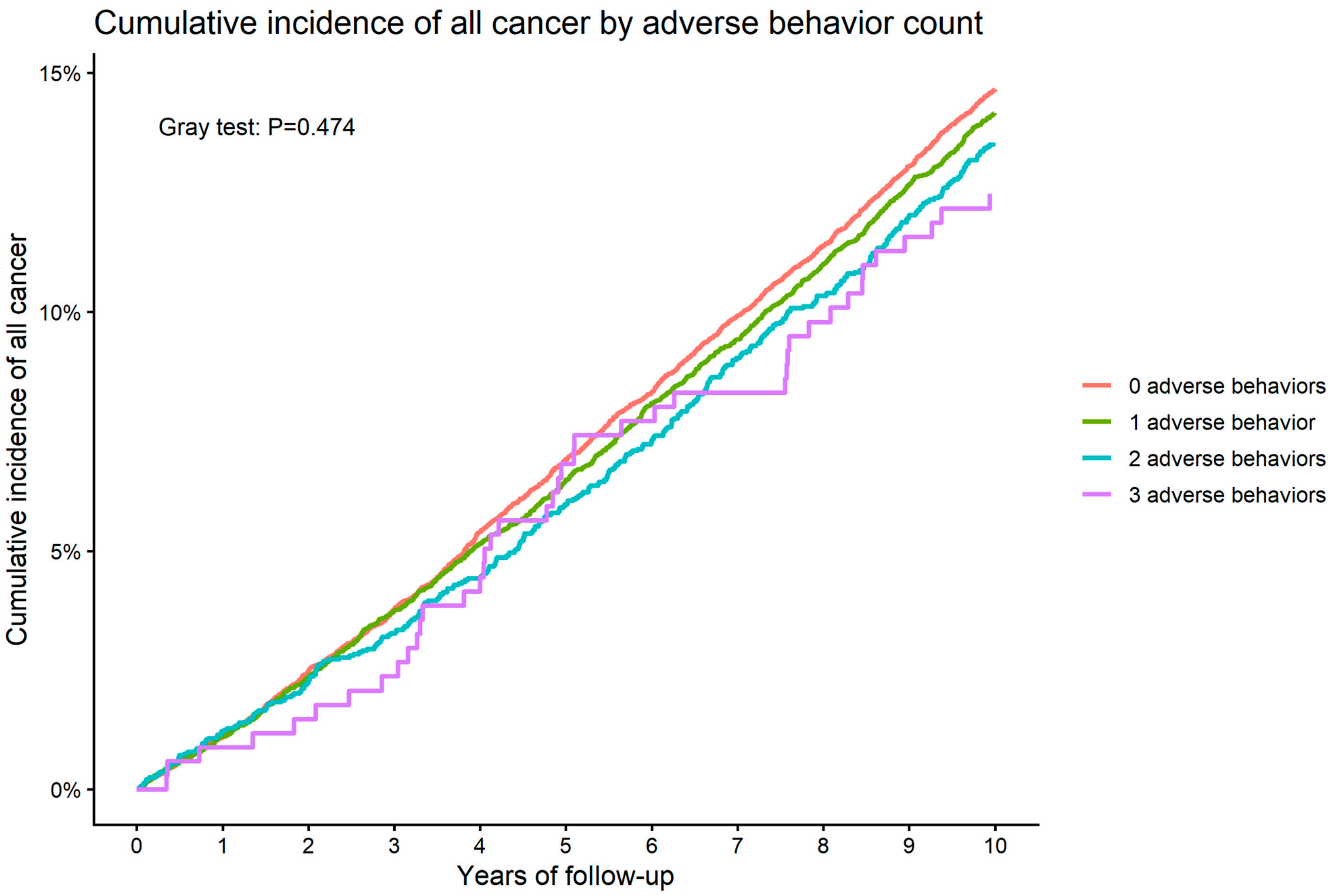

Curves were estimated using the Aalen-Johansen method with death treated as a competing event. The Gray-test P value compares cumulative-incidence functions across the four groups.

Figure S3. Cumulative incidence of all cancer according to the unified lifestyle profile.

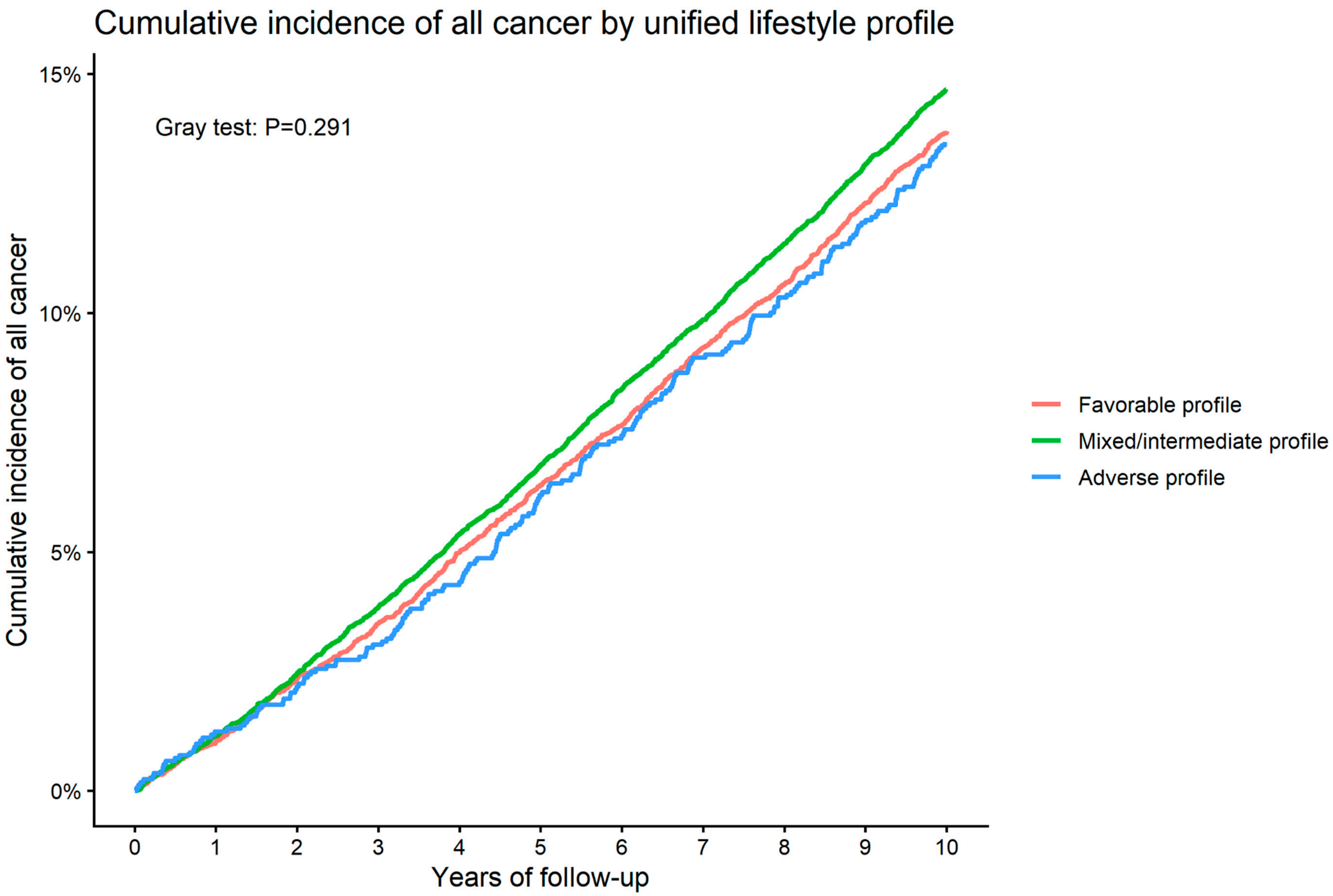

The favorable profile comprised at least two favorable behaviors and no adverse behavior; the adverse profile comprised at least two adverse behaviors and no favorable behavior. All remaining combinations were classified as mixed/intermediate.

Figure S4. Sensitivity analyses for the unified lifestyle profile.

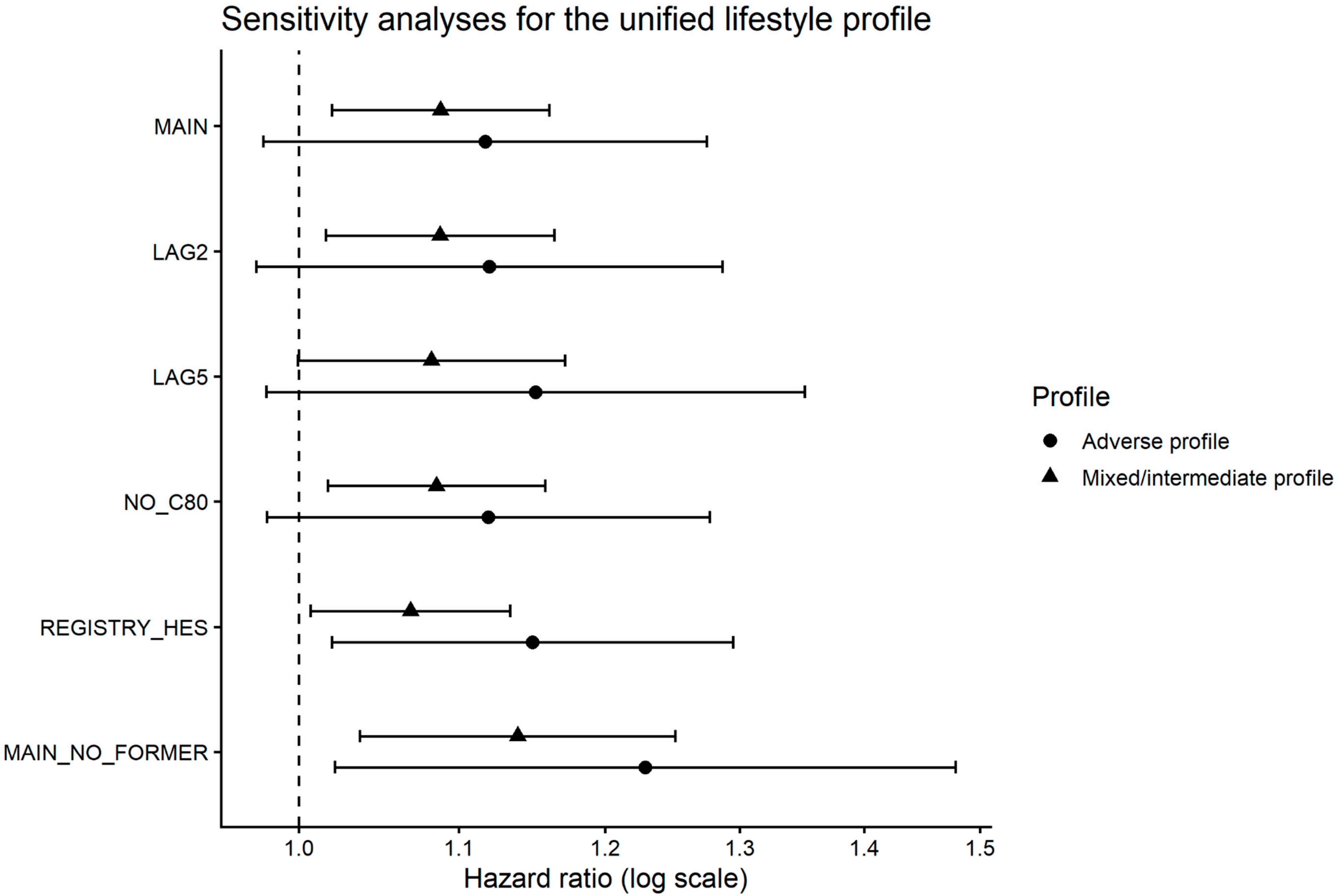

Points show adjusted hazard ratios and horizontal lines show 95% confidence intervals. The favorable profile was the reference group in every analysis.
